# Supplementary material for: Risk Factors for Delirium after Deep Brain Stimulation Surgery under Total Intravenous Anesthesia in Parkinson’s Disease Patients
Source: Brain Sci. 2022 Dec 22;13(1):25. doi: 10.3390/brainsci13010025 (PMC9856435; doi:10.3390/brainsci13010025)
Supplement: Supplementary file 1 [file brainsci-13-00025-s001.zip › brainsci-2063176-supplementary.pdf]

Supplementary Table 1. Multivariate Logistic Regression Analysis Showing the Independent Predictors of POD after Controlling for Variables with *P* below 0.2

	OR	95% CI		*P*
		Lower	Upper	
Age	1.165	0.994	1.366	0.059
Gender	0.22	0.031	1.572	0.131
BMI	1.198	0.909	1.579	0.2
ASA	0.769	0.118	4.99	0.783
Length of hospital stay	1.125	0.729	1.737	0.594
Diabetes	2.609	0.113	60.307	0.549
Operation duration	1.03	0.971	1.093	0.326
Preoperative MMSE score	0.77	0.625	0.947	0.013
Postoperative 24 h VAS pain score	1.852	0.919	3.73	0.085
Preoperative serum Na	0.915	0.53	1.581	0.75
Preoperative serum Cl	0.842	0.51	1.388	0.499
Preoperative serum glucose	1.432	0.609	3.368	0.411
UPDRS Part 1 score	0.729	0.51	1.042	0.083
UPDRS Part 2 score	1.196	0.992	1.442	0.061
UPDRS Part 3 (on state) score	1.086	1.001	1.178	0.048
UPDRS Part 3 (off state) score	0.979	0.893	1.073	0.653
NMSS score	1.401	0.962	2.041	0.079
SSA score	1.017	0.475	2.176	0.965
HAMD score	1.185	0.967	1.451	0.102
Brian hemorrhage	0.901	0.114	7.245	0.929
Brian edema	6.746	0.824	55.207	0.075

BMI, body mass index; ASA, American Society of Anesthesiologists; MMSE, Mini-Mental State Examination; VAS, visual analog scale; UPDRS, unified Parkinson's disease rating scale; NMSS, non-motor symptom scale; SSA, standardized swallowing assessment; HAMD, hamilton depression scale; POD, postoperative delirium; OR, odds ratio; CI, confidence interval.
